# Submucosal tunneling endoscopic resection with bidirectional full-thickness resection of gastrointestinal stromal tumor: a case report

**DOI:** 10.1016/j.igie.2025.03.009

**Published:** 2025-04-01

**Authors:** Farimah Fayyaz, Preethi Jagannath, Jose Antonio Almario, Mouen A. Khashab

**Affiliations:** 1 Johns Hopkins Medicine, Baltimore, Maryland, USA; 2 Division of Gastroenterology, Department of Medicine, Chulalongkorn University, Bangkok Thailand

## Abstract

Gastrointestinal stromal tumors (GISTs) are the most common mesenchymal neoplasms of the gastrointestinal tract. Whereas surgical resection remains the criterion standard for high-risk lesions, minimally invasive endoscopic techniques are emerging as viable alternatives. Exophytic subepithelial lesions pose unique challenges for standard endoscopic techniques, including incomplete resection and potential injury to adjacent structures. This case report presents a novel bidirectional full-thickness resection (FTR) technique for resecting an exophytic gastric GIST in a 63-year-old man. The procedure involved submucosal tunneling, peritoneal entry, and circumferential FTR from both the peritoneal and the tunnel sides, ensuring complete removal of the lesion. Mucosal incision closure was achieved by use of an endoscopic suturing system, with no postprocedural adverse events. The diagnosis of GIST was confirmed histopathologically. Twenty-one months after the procedure, no recurrence was observed on imaging. Bidirectional FTR potentially enhances visualization, improves resection completeness, and minimizes procedural risks, making it a promising technique for managing exophytic subepithelial lesions.

Gastrointestinal stromal tumors (GISTs) are the most prevalent mesenchymal neoplastic subepithelial lesions in the gastrointestinal (GI) tract, the stomach being the most frequent primary tumor site.^1^ They are often identified incidentally during endoscopy as subepithelial lesions and require further evaluation to determine their potential for malignancy.

Surgical resection remains the criterion standard for GIST management, particularly for lesions >2 cm or those exhibiting high-risk features. However, minimally invasive endoscopic techniques are emerging as promising alternatives. They include submucosal tunneling endoscopic resection (STER), full-thickness resection (FTR), and endoscopic submucosal dissection (ESD).^2^

Exophytic subepithelial lesions pose unique challenges for standard endoscopic techniques, such as incomplete resection, injury to adjacent structures, and potential bleeding.^3^ This case report introduces a modified endoscopic FTR technique, termed bidirectional FTR, specifically designed to address those challenges in the resection of exophytic lesions. Bidirectional FTR refers to visualizing and resecting the lesion from both the luminal/submucosal and the peritoneal sides.

## Case presentation and endoscopic methods

A 63-year-old man with no significant medical, family, or social history underwent multiple unremarkable upper endoscopies because of abdominal pain. Most recently, abdominopelvic computed tomography with contrast medium revealed a homogenous 1.7 × 1.2 cm partially exophytic nodule along the distal greater curvature of the stomach, suggestive of GIST. The result of the physical examination was unremarkable, with no abdominal tenderness or distension. More than 2 months after the lesion was identified, the patient underwent an endoscopic procedure for its removal. The decision to proceed with endoscopic resection was driven by the patient's preference for intervention over surveillance.

**Figure 1:**
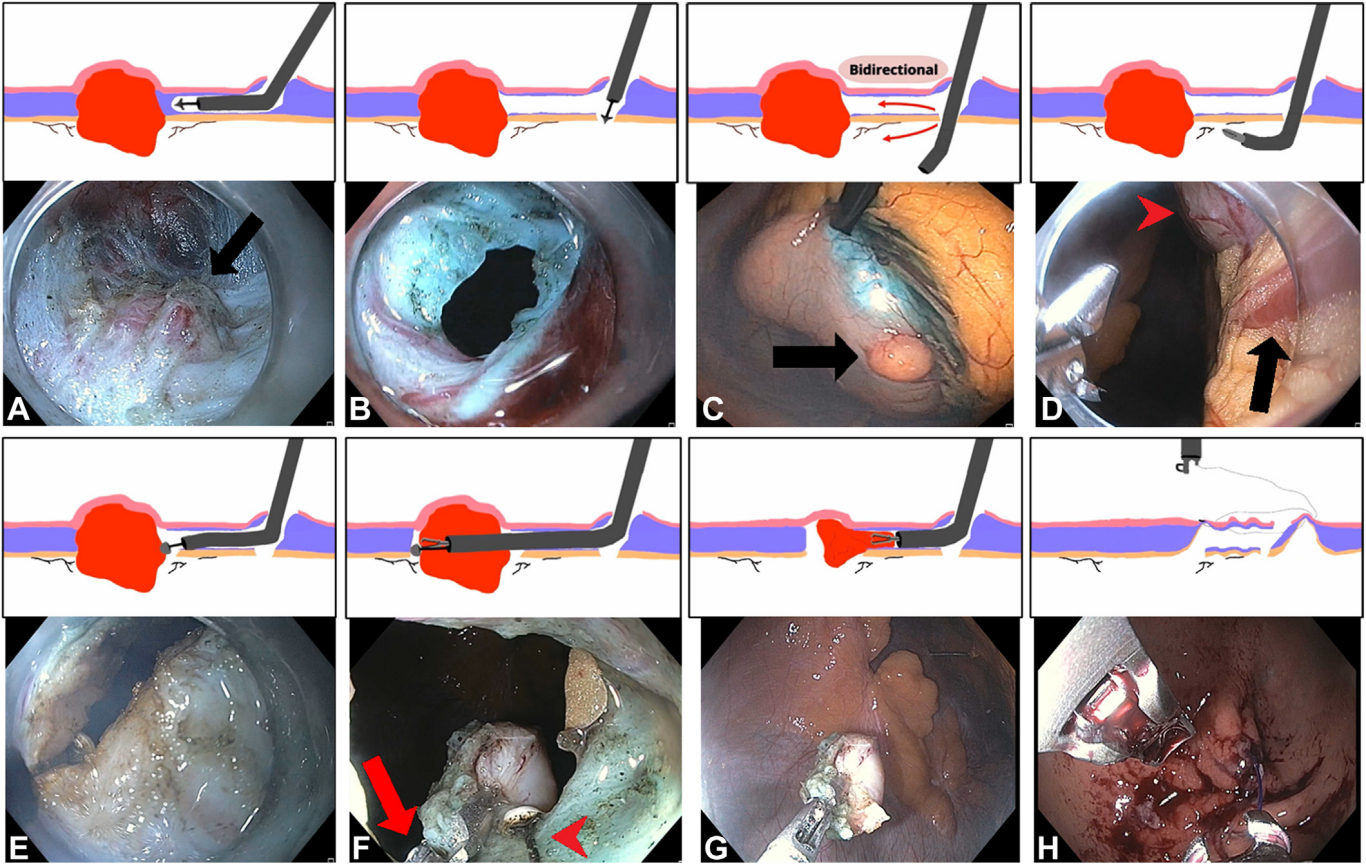
Submucosal tunneling endoscopic resection with bidirectional full-thickness resection of GI stromal tumor. **A,** Submucosal dissection and tunneling. Cartoon shows black endoscope in purple submucosal tunnel approaching irregular red exophytic subepithelial lesion. Endoscopic image inside submucosal tunnel showing exophytic lesion (*arrow*). **B,** Myotomy and entering peritoneum. Cartoon shows black endoscope performing myotomy proximal to irregular red lesion to enter peritoneum. Endoscopic image showing opening into peritoneum. **C,** Peritoneal view of lesion. Cartoon shows black endoscope in peritoneal cavity. Endoscopic view inside peritoneal cavity with visualization of exophytic portion of lesion (*arrow*). **D,** Coagulation of gastric wall vessels around lesion. Cartoon shows black endoscope with coagulation forceps treating vessels (*irregular black lines adjacent to red lesion*). Endoscopic image showing blood vessels (*arrow*) adjacent to lesion (*arrowhead*). **E,** Circumferential full-thickness resection of lesion. Cartoon shows black endoscope back in purple submucosal tunnel and performing resection. Endoscopic image shows use of endoscopic submucosal dissection knife. **F,** Grasping lesion and completing resection. Both cartoon and endoscopic image show double-channel gastroscope with forceps (*arrow*) in 1 channel grasping lesion and endoscopic submucosal dissection knife (*arrowhead*) in second channel completing resection. **G,** Lesion retrieval. **H,** Mucosal incision closure with endoscopic suturing device.

The subepithelial lesion found in the distal part of the gastric body, along the greater curvature, was first marked with soft coagulation to guide the submucosal dissection (Fig. 1, Video 1, available online at www.igiejournal.org). The mucosal incision was created along the greater curvature in the mid gastric body, 7 cm proximal to the lesion. A submucosal injection, followed by mucosal incision, was performed, and the submucosal fibers were dissected. The submucosal tunnel was further extended until the subepithelial lesion was fully exposed. The lesion was noted to be primarily exophytic, and the decision was made to proceed with FTR. Proximal to the lesion in the submucosal tunnel, cautery was applied to the muscle, and a blunt passage was used to create entry into the peritoneum. The exophytic component of the mass was identified from the peritoneal side, and gastric wall vessels around the lesion were identified and coagulated. A circumferential FTR of the lesion was then performed from both the peritoneal and the tunnel sides. Before completion of the resection, the gastroscope was exchanged for a double-channel gastroscope. The lesion was securely grasped by forceps, an ESD knife was passed through the second channel, and FTR of the lesion was completed while a firm grasp of the lesion was maintained. The lesion was then successfully retrieved. Complete closure of the mucosal incision site was achieved by use of an endoscopic suturing system. On day 1 after the procedure, an upper GI series showed no evidence of leakage. No postprocedural adverse events were observed, and the diagnosis of GIST with negative margins was confirmed histopathologically. The patient was discharged 2 days after the procedure and expressed satisfaction with the minimally invasive approach for lesion removal. At his most recent follow-up visit, 21 months after the procedure, magnetic resonance imaging demonstrated no recurrence. Despite successful removal of the lesion, the patient’s abdominal pain persisted and was not attributed to the GIST. Further evaluation for the abdominal pain is ongoing.

## Conclusion

Bidirectional FTR appears to offer a safe and minimally invasive approach for managing exophytic subepithelial lesions. The addition of STER, similar to a peroral endoscopic myotomy tunnel, provides controlled access and can facilitate closure by preserving the mucosa. Alternatively, standalone bidirectional FTR can be performed by creating an incision proximal to the lesion, followed by submucosal dissection and the application of traction to delineate its borders before completing the FTR.^4^ This case report describes a single-patient experience and therefore may not be generalizable to all exophytic GISTs or subepithelial lesions in varied anatomic locations. Additionally, whereas the long-term follow-up time of 21 months without recurrence is encouraging, larger studies with longer surveillance periods are needed to confirm the durability and safety of bidirectional FTR. Operators’ expertise may also limit the widespread adoption of this technique. Nevertheless, bidirectional FTR appears to not only reduce the risk of incomplete resection but also to enhance dissection, provide an additional dimension of visualization, and minimize the risk of injury to surrounding structures and blood vessels. Further experience with this technique and long-term follow-up times are needed.

## Disclosure

The following author disclosed financial relationships: M. A. Khashab: consultant for Boston Scientific, Medtronic, and Olympus America, and recipient of royalties from UpToDate and Elsevier. All other authors disclosed no financial relationships.
